# Activation of the CCL22/CCR4 causing EMT process remodeling under EZH2-mediated epigenetic regulation in cervical carcinoma

**DOI:** 10.7150/jca.101881

**Published:** 2024-10-14

**Authors:** Li Zhang, Sijuan Tian, Jie Chang, Shimin Quan, Ting Yang, Minyi Zhao, Li Wang, Xiaofeng Yang

**Affiliations:** Department of Gynecology and Obstetrics, the First Affiliated Hospital of Xi'an Jiaotong University, No.277 West Yanta Road, Xi'an 710061, China.

**Keywords:** cervical cancer, EZH2, DNMT3A, CCL22-CCR4, EMT

## Abstract

Cervical cancer (CC) is an important public health problem for women, gene expression patterns which were governed by epigenetic modifications can result in CC, CC-chemokine receptor 4 (CCR4) interacts with C-C-motif ligand 22 (CCL22) is associated with tumor progression or metastasis. A previous study by the present authors revealed the levels of chemokine CCL22 and its receptor CCR4 are increased in CC tissues, nevertheless, the regulatory mechanisms governing its expression remain poorly understood. The present study aimed to investigate the potential role of enhancer of zeste homolog 2 (EZH2)-induced epigenetic activation of CCL22/CCR4 and caused epithelial-to-mesenchymal transition (EMT) remodeling in CC. CCL22 and CCR4 were significantly up-regulated in CC samples compared with normal cervix tissues, and obvious induction of promoter DNA methylation levels of *CCL22* and *CCR4* was found in CC tissues. Demethylation reactivated the transcription of *CCL22* and *CCR4*. DNA methyltransferase 3A (DNMT3A) was found to directly bind to the *CCL22* and *CCR4* promoter regions *in vitro*. Downregulation of the expression of EZH2 in CC cell lines altered DNMT3A expression and induced *CCL22* and *CCR4* promoters' methylation levels, while *CCL22* and *CCR4* mRNA expression decreased. An *in vivo* assay showed that EZH2 regulated the expression of CCL22/CCR4 components through DNMT3A, consistent with the *in vitro* results. In EZH2-silenced CC cells, migration was reduced, levels of EMT-related markers, including vimentin, slug, snail and β-catenin, were all reduced and zona occludens 1 (ZO-1) increased. In DNMT3A-silenced CC cells, migration was induced, vimentin, slug, snail and β-catenin were all induced and ZO-1 was reduced. Inhibition of CCL22 protein significantly decreased migration of CC cells and vimentin, slug, snail and β-catenin levels, while ZO-1 increased. In conclusion, EZH2 appears to regulate CCL22/CCR4 expression via epigenetic activation, causing EMT process remodeling in CC progression.

## Introduction

Cervical cancer (CC) ranks as the fourth most common female malignancy and represents a major global health burden. In 2020, there were ~604,127 new cases and 341,831 deaths because of CC 1. In 2022, China was projected to see 150,700 new cases of CC and 55,700 related fatalities 2.

The epigenetic regulation of genes, including histone modifications and DNA methylation, is important in regulating gene transcription. DNA methylation is often associated with changing gene expression in cancer. Alterations in histone modification states could accelerate alterations in DNA methylation, indicating that alterations may promote the recruitment of DNA methyltransferases 3. DNA methylation is highly correlated with gene silencing 4 and is established by the specialized *de novo* DNA methyltransferase enzyme methyltransferase 3A (DNMT3A) 5-7. Enhancer of zeste homolog 2 (EZH2) is a core component of polycomb repressive complex 2 (PRC2) that induces silencing of target genes 8. A previous study by the present authors revealed that EZH2 induced the expression of methylation of histone H3 lysine 27 (H3K27me3), which consequently reduced the expression of DNMT3A, causing changes in the expression of the downstream immune factors T cell immunoglobulin domain and mucin domain-3 (Tim-3), and galectin-9 9. Moreover, it showed that EZH2-mediated methylation regulation plays an important role in the expression of immune factors in the cervical tumor microenvironment and promotes tumorigenesis.

Epithelial-to-mesenchymal transition (EMT) is a cellular process in which cells lose their epithelial characteristics and acquire mesenchymal features, which enable them to migrate more efficiently and invade the underlying mesenchyme. In cancer, EMT is associated with tumorigenesis, invasion, metastasis and resistance to therapy 10. It has been reported that the H3K27me3 was a master regulator of EMT through the methyltransferase EZH2 11.

Previous research performed by the present authors found that the expression of negative immune regulators such as Tim-3/galectin-9 and C-C-motif ligand 22 (CCL22)/CC-chemokine receptor 4 (CCR4) exceeds that of the positive immune factors in the CC microenvironment, giving rise to immune disequilibrium and contributing to the progression of cervical carcinogenesis 12-14. CCL22 is a chemokine and is known to be a negative immune factor. CCL22 is the ligand for CCR4, which is mostly expressed on the surface of T helper 2 cells (Th2) cells and regulatory T cells (Tregs). CCL22 is highly expressed in several types of tumors and is known to recruit Tregs into tumor tissue. Nonetheless, the regulatory mechanisms of CCL22 expression in cancer tissues are poorly understood thus far 15, 16.

The current study showed that EZH2-based epigenetic modifications play critical roles in regulating the expression of CCL22 and CCR4. Interestingly, high CCL22 and CCR4 expression levels showed significantly increased EMT potential and migration, and down-regulation of EZH2 could suppress EMT and migration of CC cells. Overall, the present study elucidated that EZH2 participates in regulating EMT and metastasis via a novel EZH2/H3K27me3/DNMT3A-CCL22/CCR4 pathway that could potentially be relevant in regulating aggressiveness in CC. This regulatory mechanism of EZH2 and CCL22/CCR4 could provide evidence and clues for developing novel therapeutic targets to suppress the progression of CC.

## Material and methods

### Tissue samples

A total of 32 cervical cancer tissues and 32 normal cervix tissues were selected for this study (Table 1 and 2), patients with stage I-III. The average age of the cervical cancer patients recruited was 45.1 ± 10.6 years (age range, 27‑68 years), the average age of the normal patients recruited was 46.4 ± 6.6 years (age range, 27‑66 years). The samples were obtained from the First Affiliated Hospital of Xi'an Jiaotong University between January 2021 and December 2022.Tumor samples and normal cervix samples were collected during surgery, and the excised samples were transferred to a sterile petri dish, where necrotic tissue and blood on the surface were washed with pre-cooled sterilized phosphate buffered saline (PBS) for 2-3 cycles, before being placed in a cryogenic storage tube for quick-frozen in liquid nitrogen and stored at -80°C until use. All tumor patients were diagnosed by two senior pathologists and none had received chemotherapy or radiotherapy before surgery. The control group underwent total hysterectomy due to benign gynecological conditions, with no cervical lesions identified in postoperative pathological examinations.

### Bioinformatics analysis

Gene Expression Profiling Interactive Analysis (GEPIA) database (http://gepia.cancer-pku.cn/) was used to detect the *CCL22* and *CCR4* mRNA expression levels in CC and normal cervix tissues, the correlation between *CCL22* and *CCR4* expression were also studied by the GEPIA database, GEPIA data are derived from integrated the Cancer Genome Atlas (TCGA) and the Genotype-Tissue Expression (GTEx) gene expression information.

### Cell culture

SiHa, HeLa and C33A cell lines were purchased from the Cell Bank, Shanghai Institutes for Biological Sciences, Chinese Academy of Sciences, Shanghai. The tumor cells were cultured in Dulbecco's minimum essential medium (DMEM) (HyClone, USA) and supplemented with 10% fetal bovine serum (FBS) (Biological Industries, Israel), in a humidified 37℃ incubator containing 5% CO_2_. The medium was replenished every 2 days and the cells were passaged every 3 days.

### Plasmid transfection

Transient and stable transfections with plasmids were according to the Lipofectamine 2000 transfection reagent (Invitrogen, USA) manufacturer's instructions. The short-hairpin (sh)RNA against *EZH2* gene (PGPU6/GFP/Neo-EZH2-homo-488) and corresponding control shRNA (GenePharma, Shanghai) were used for RNA interference. The gene silencing effects were confirmed by western blotting at 48 h after transfection. Stably transduced cells were maintained in culture in the presence of geneticin (G418).

### Transfection of siRNA into cells

The siRNA oligonucleotides (GenePharma, Shanghai), targeted to EZH2 or DNMT3A, were used to knock-down EZH2 or DNMT3A. In a 6-well plate, SiHa and HeLa cells were seeded to 50%-60%. Then, cells in were transfected with siRNA specific for EZH2 or DNMT3A with Lipofectamine 2000 (Invitrogen, USA) according to the manufacturer's instructions. In parallel, scrambled siRNA was used as a control for off-target changes in SiHa and HeLa cells. Twenty-four hours after transfection, the medium was changed and cells were incubated for an additional 24 h before being harvested for analysis. The primers used in the following siRNA oligos are listed in Table 3.

### CCL22 and DZNep treatment

Recombinant human CCL22 (NBP2-34977, NOVUS, USA) was reconstituted according to instructions provided by the manufacturer. Treatment in this study was done using a concentration of 100 ng/ml, 200 ng/ml, the data were collected at 24 h time point. The EZH2 inhibitor 3-Deazaneplanocin A (DZNep) was provided by (Selleck, USA). Stock solution was dissolved in sterilized ddH_2_O and tested at 5 μM and 10 μM for 48 h.

### Neutralization of CCL22 in CC cells

SiHa and HeLa cells were suspended at a density of 1 × 10^6^ cells/ml in a dilution medium containing 1.5 μg/ml and 3 μg /ml of Neutralization of CCL22 antibody (MAB336-100, R&D, USA) per ml and pre-incubated at 37 °C for 24 h.

### Quantitative real-time PCR (RT-qPCR) assays

Total RNA was collected using TRIzol reagent (Sigma, USA) as previously 9, in brief, cDNAs were synthesized using the PrimeScript RT Reagent Kit (Taraka, China) according to the manufacturer's instructions. RT-qPCR was conducted in triplicate with LightCycler2.0 (Bio-Rad, USA), and the expression was normalized to GAPDH as the total RNA and cytoplasmic RNA endogenous control. PCR primers primer sequences are listed in Table 3. The relative quantification value for each target gene was expressed as 2^-△△Cq^.

### 5‑Aza‑2'‑deoxycytidine (5-Aza-CdR) treatment, DNA extraction, bisulfite modification

5-Aza-CdR (Sigma, USA) was reconstituted in complete DMEM to final concentrations of 5 μM, 10 μM indicated in results. Fresh treatment of 5-Aza-CdR was given to cells daily over 72 h prior to assaying. Genomic DNA of CC cells and tissues were extracted by TaKaRa Mini BEST Universal Genomic DNA Extraction kit (TaKaRa, China) according to the manufacturer's instructions. 500 ng of genomic DNA was bisulfite-modified using EZ DNA Methylation-Gold™ kit (Zymo Research, USA).

Methylation-specific PCR (MS-PCR) and Quantitative Methylation-specific PCR (MS-qPCR) modified DNA templates were used for MS-PCR with Zymo TaqTM PreMix (E2003, Zymo Research, USA) following the instructions. The annealing temperature for the methylated CCL22-CCR4 primer was 55°C and for the unmethylated primer was 55°C. The MS-PCR products were separated on 2% agarose gel, stained with Gelview and visualized under ultraviolet illumination (Bio-Rad, USA). Methylation level was calculated by the ratio of methylated and unmethylated levels. Grey value of each band represented its relative expression and was measured by Image J Software. The MS-qPCR was operated with TB Green® Premix Ex Taq™ II (TaKaRa, China) by a two-step amplification procedure according to the manufacturers' protocol. For each sample, a relative methylation level was calculated using the difference in Cq values by the standard 2^-△△Cq^ method in which *ALU* was used as an internal reference gene. The primers used in the MS-PCR and MS-qPCR are listed in Table 3.

### Western blotting

Cells were scraped into radio immunoprecipitation assay (RIPA) lysis buffer. Proteins were running to separate on sodium dodecyl sulfate (SDS) polyacrylamide gels, electrophoretic ally transferred to polyvinylidene fluoride (PVDF) membranes, and incubated with primary antibodies. The relative protein expression of each band was normalized to β-actin or GAPDH, and the normalized ratio of the control group was set as 1 17. The primary antibodies are listed in Table 4. Proteins were visualized with second antibody. The secondary antibodies are as follows: HRP-conjugated rabbit anti-mouse IgG (1:5,000 dilution, D110273-0100, BBI Life Sciences, China), HRP-conjugated goat anti-rabbit IgG (1: 5,000 dilution, 31460, PIONEER, China).

### Chromatin immunoprecipitation (ChIP) assay

The ChIP experiments were executed using the Simple ChIP Enzymatic Chromatin IP Kit (Cell Signaling Technology, USA) as previously 18, in briefly, 1×10^7^ cells were cross-linked with 1% formaldehyde for 10 minutes at room temperature. The crosslinking was then quenched with 10×glycine. Chromatin was sonicated in lysis buffer to 200-1,000 bp, and the extraction of ChIP DNA was performed as per the kit's protocol. The primer sequences are given in Table 3. The antibodies utilized included EZH2, H3K27me3 and DNMT3A (show in Table 4).

### Tumor xenograft experiments

24 cohorts of 4-week-old female BALB/c-nu mice and weighing between 16‑18 g were divided into 4 groups. The mice were housed in specific pathogen‑free conditions with 24‑26˚C temperature, 50‑60% humidity and 12/12‑h light/dark cycle at the Laboratory Animal Center, Xi'an Jiaotong University Health Science Center, where they were provided with autoclaved water and food ad libitum. EZH2 knocked-down SiHa and HeLa cells (1×10^7^ cells per ml) were resuspended in 100 µl PBS and injected subcutaneously into the flank of the nude mice. Engrafted mice were monitored for tumor development through visual inspection until tumor formation occurred. Every 2 days tumors were checked once palpable, mice were monitored for 15 days, tumor growth and mouse weight were monitored until death. Tumor volume was calculated as previously described 18, using the following formula: tumor volume (mm^3^) =0.5×length×width^2^. The mice were euthanized and tumors and organs were extracted. Mice were administered 2.5% isoflurane for induction of anesthesia by inhalation for 3 min and then 1.5% isoflurane for maintenance of anesthesia before being euthanized. Mice were euthanized by cervical dislocation under anesthesia to ensure humane conditions for the study. The mice were observed to have no response, including limb paralysis and no rise and fall of the chest to confirm death.

### Transwell chamber migration assay

5 × 10^4^ cells were resuspended in 200 μl of serum-free medium per sample. Cell suspension was transferred to the upper chamber of the transwell (BD, Franklin Lakes, NJ) for migration through 8.0 μm pore polyethylene terephthalate (PET) membrane in a 24-well plate setting. Migrating cells were fixed with formalin followed by staining with crystal violet dye (0.1%). The stained membrane was then washed and photographed using a light microscope. Five images per chamber were taken from random fields of view and migration was quantified as average area occupied by migrated cells using Image J software.

### Statistical analyses

Statistical analyses were performed using GraphPad Prism 8 software (GraphPad Software, USA). A paired t‑test and one‑way analysis of variance (ANOVA) were carried out on samples within groups, Dunnett's test was used as the post hoc test after one‑way ANOVA. The data are presented as the means ± standard error of the mean (SEM). All experiments were independently repeated at least thrice, with consistent results.

## Results

### Promoter DNA hypomethylation contributes to the overexpression of CCL22 and CCR4 in CC

**Figure 1:**
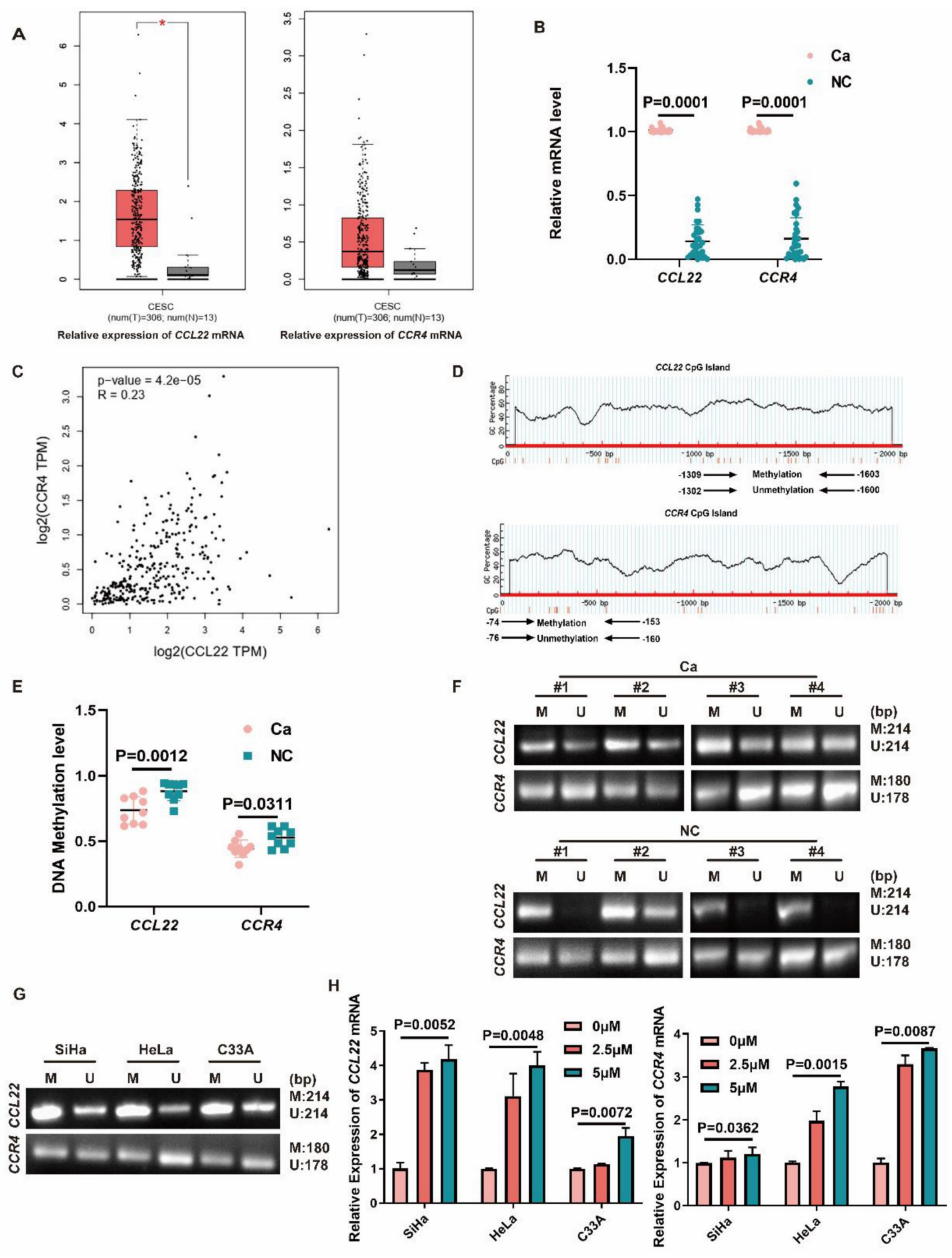
** Hypomethylation states of *CCL22* and* CCR4* caused overexpression of *CCL22* and *CCR4* in CC**. (**A**) An overview of mRNA levels of *CCL22* and *CCR4* in CC based on GEPIA database. (**B**) The mRNA levels of *CCL22* and *CCR4* in CC (Ca) (n=32) and normal cervical tissues (NC) (n=32) detected by RT-qPCR. (**C**) The correction between *CCL22* and *CCR4* in CC, analyzed by GEPIA database. (**D**) Predicted CpG islands in the promoter regions of *CCL22* and *CCR4*. Numbers indicate the positions in bp relative to the transcription start site. The blue region represents the CpG islands and the red vertical bars are the CpG loci in these input sequences. (**E** and **F**) DNA Methylation level of *CCL22* and *CCR4* promoter regions in CC (Ca) (n=9) and NC (n=9) detected by MS-PCR. MS-PCR images of 4 representative samples are shown from each group. (**G**) Detection of *CCL22* and *CCR4* promoter DNA methylation status by MS-PCR in SiHa, Hela and C33A cells; (M: methylated, U: unmethylated). (**H**) Relative mRNA expression of *CCL22* and *CCR4* in SiHa, HeLa and C33A cells after treatment with different concentrations of 5-Aza-CdR.

*CCL22* mRNA expression was higher in CC tissues than normal tissues according to the GEPIA database (num(T)=306, num(N)=13) (Fig. 1A) (http://gepia.cancer-pku.cn/). The results of qPCR analysis of 32 CC tissues and 32 normal cervix tissues demonstrated that *CCL22* and *CCR4* expression was significantly increased in CC samples (Fig. 1B) (Table 3). The expression levels of *CCL22* and *CCR4* were correlated in the GEPIA dataset (r = 0.23, *P* = 0.000042) (Fig. 1C), indicating that these genes might partially share common biological functions.

MS-PCR results showed that the promoter DNA methylation levels of *CCL22* and *CCR4* were downregulated in CC tissues (Fig. 1D, E and F), demonstrating that promoter DNA hypomethylation states of *CCL22* and *CCR4* are associated with upregulated expression of CCL22-CCR4 in CC samples.

In Fig. 1G, SiHa, HeLa and C33A cells all showed partial DNA methylation in the *CCL22*-*CCR4* promoter regions. As shown in Fig. 1H, after treatment with 5-Aza-CdR (0, 5, 10 μM) for 72 h, *CCL22* and *CCR4* mRNA levels increased in a dose-dependent manner. Demethylation of *CCL22*-*CCR4* reversed these expression changes.

### DNMT3A enrichment at the promoter site caused CCL22-CCR4 hypomethylation

**Figure 2:**
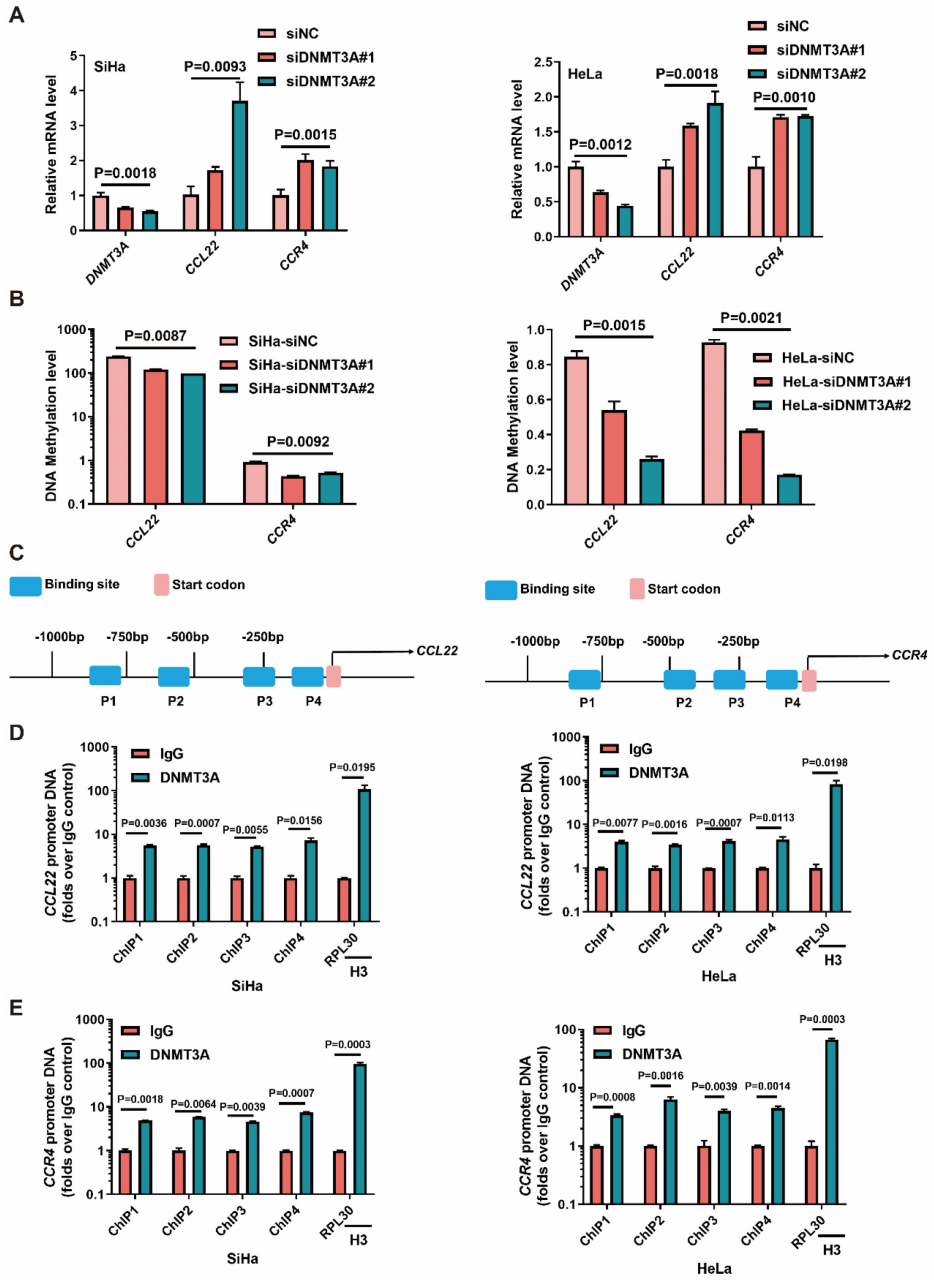
** DNMT3A reactivated the *CCL22* and *CCR4* expression through decreasing promoters' DNA methylation**. (**A**) Detection of the expression of *DNMT3A*, *CCL22* and *CCR4* in DNMT3A specific siRNA transfected SiHa and HeLa cells by RT-qPCR. (**B**) Detection of the DNA methylation level of *CCL22* and *CCR4* in DNMT3A specific siRNA transfected SiHa and HeLa cells by MS-qPCR. (**C**) Schematic representation of the 4 regions of the *CCL22* and *CCR4* promoter regions amplified in the chromatin immunoprecipitation (ChIP)‑quantitative PCR (qPCR) experiment. (**D** and **E**) Chromatin was cross-linked, fragmented and immunoprecipitated with either IgG (mock) or anti-DNMT3A ChIP-grade antibody and the purified DNA was used to amplify with respective primer pairs for indicated four regions in the *CCL22* and *CCR4* promoter regions in qPCR. The enrichment of DNMT3A on *CCL22* and *CCR4* promoter regions relative to IgG in SiHa and HeLa cells, and H3 against RPL30 was used as positive control.

We next examined whether the level of DNMT3A determined the expression of CCL22 and CCR4 in SiHa and HeLa cells. The *CCL22* and *CCR4* mRNA levels were increased in SiHa and HeLa cells with DNMT3A knocked-down (Fig. 2A). The MS-qPCR results showed that the DNA methylation levels of *CCL22* and *CCR4* decreased after knockdown of DNMT3A expression (Fig. 2B). Thus, we hypothesized that DNMT3A could bind to the *CCL22* and *CCR4* promoters in CC cells (Fig. 2C). To investigate the potential interaction between DNMT3A and CCL22-CCR4, a ChIP-qPCR assay was carried out. The results showed that DNMT3A was significantly enriched in the *CCL22* (Fig. 2D) and *CCR4* (Fig. 2E) promoter regions in SiHa and HeLa cells (IgG was used as the control antibody). It was indicated that *CCL22* and *CCR4* were targets of DNMT3A.

### EZH2 leads to the epigenetic regulation of DNMT3A in CC, causing CCL22-CCR4 hypomethylation

**Figure 3:**
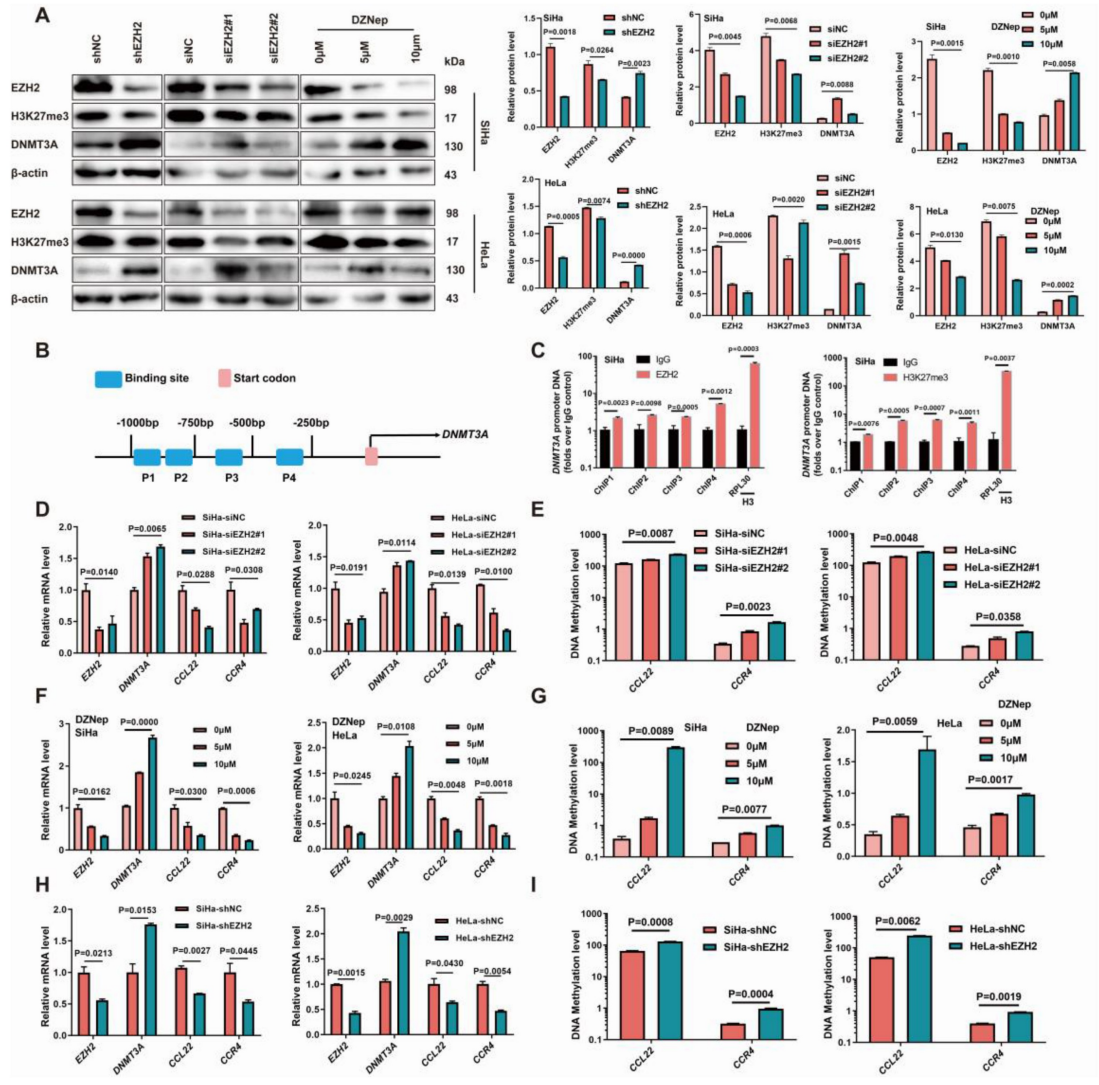
** Inhibition of EZH2 promoted DNMT3A in cervical cancer cells with methylated the promoter regions of *CCL22* and *CCR4***. (**A**) Detection of the expression of EZH2, H3K27me3 and DNMT3A in EZH2 knocked-down or specific siRNA transfected or DZNep treated SiHa and HeLa cells by western blotting. (**B**) Schematic representation of the 4 regions of the *DNMT3A* promoter region amplified in the chromatin immunoprecipitation (ChIP)‑quantitative PCR (qPCR) experiment. (**C**) Chromatin was cross-linked, fragmented and immunoprecipitated with either IgG (mock) or anti-EZH2 and H3K27me3 ChIP-grade antibody and the purified DNA was used to amplify with respective primer pairs for indicated four regions in the *DNMT3A* promoter region in qPCR. The enrichment of EZH2 and H3K27me3 on *DNMT3A* promoter region relative to IgG in SiHa cell, and H3 against RPL30 was used as positive control. (**D**, **F** and **H**) The mRNA expression of *EZH2*, *DNMT3A* and *CCL22*-*CCR4* in EZH2 knocked-down or specific siRNA transfected or DZNep treated SiHa and HeLa cells by RT-qPCR. (**E**, **G** and **I**) Detection of the methylation level of *CCL22* and *CCR4* in EZH2 knocked-down or specific siRNA transfected or DZNep treated SiHa and HeLa cells by MS-qPCR.

EZH2 was knocked down using EZH2 siRNAs and shRNA in SiHa and HeLa cell lines. The H3K27me3 levels decreased and the DNMT3A protein levels increased after EZH2 knocked-down (Fig. 3A). The mRNA levels of *CCL22* and *CCR4* were decreased, and the promoter DNA methylation levels eventually increased in the EZH2-downregulated groups (Fig. 3D, E, H and I). When SiHa and HeLa cells were treated with the EZH2 inhibitor DZNep, a marked reduction in H3K27me3 levels was observed, and the DNMT3A level after DZNep treatment was significantly increased (Fig. 3A), meanwhile, mRNA levels of *CCL22* and *CCR4* were significantly decreased, and their methylation levels increased accordingly (Fig. 3F and G). The ChIP results also showed that EZH2 and H3K27me3 were remarkable enriched in the *DNMT3A* promoter region (Fig. 3B and C) 9. These results suggested that EZH2-mediated DNA methylation through H3K27 trimethylation changing could attribute to the higher expression level of CCL22-CCR4 in CC cells.

### EZH2-mediated the migration and EMT remodeling of CC cells through CCL22-CCR4

**Figure 4:**
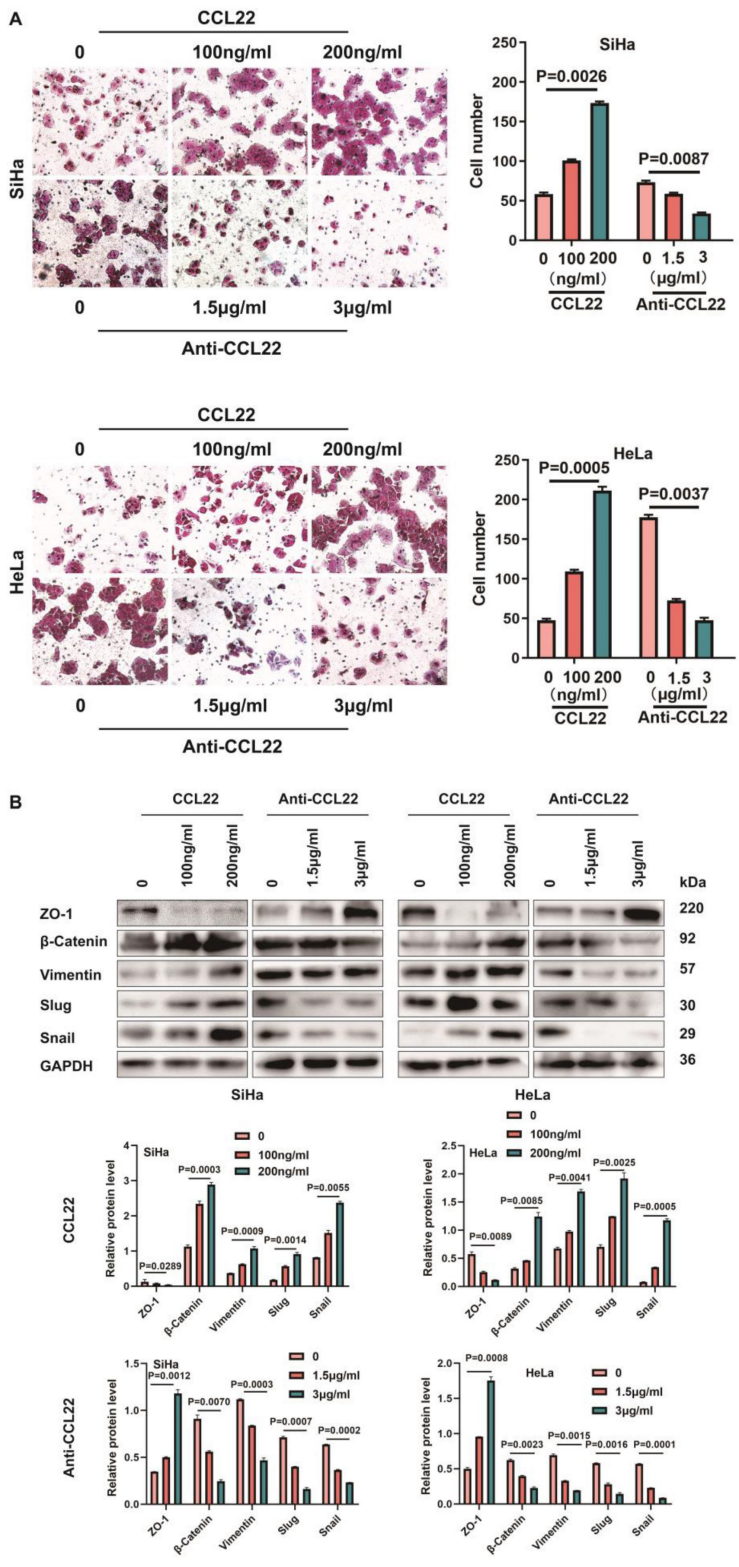
** Effect of CCL22-CCR4 on migration of CC cells**. (**A**) The migratory potential of SiHa and HeLa cells which added recombinant human CCL22 protein or neutralization CCL22 antibody and the respective control cells was analyzed by the transwell cell migration assay. Number of migratory cells was shown as means ± standard error from three independent experiments using triplicate measurements and statistically analyzed with Student's t-test in each experiment. Magnification, ×200. (**B**) The expression of EMT-related proteins in SiHa or HeLa cells which added recombinant human CCL22 protein or neutralization CCL22 antibody and the respective control cells was determined by western blotting and the gray level analysis of the protein levels of EMT-related proteins.

CCL22 promotes stemness of cancer cells with CCR4 expression, causing cancer cells migration and EMT, as shown on many types of cancers 19. The transwell chamber assay clearly revealed that CC cells treatment with 100 ng/ml and 200 ng/ml recombinant human CCL22 could increase the migration ability and neutralization with the CCL22 antibody reversed CCR4-expressing CC cell migration augment (Fig. 4A), indicating that CCL22 secreted by cervical cancer cells bound with CCR4 which expressed on the surface of cancer cells could promote CC cells migration, we assessed the effect of CCL22 bound with CCR4 on a few essential EMT markers, vimentin, Zona Occludens 1 (ZO-1) and slug, snail and β-catenin. Interestingly, cells treated with recombinant human CCL22 exhibited increased vimentin, slug, snail and β-catenin levels and decreased ZO-1 levels, neutralization with the CCL22 antibody caused the opposite effect (Fig. 4B), indicating CCL22 stimulation contribute CCR4-expressing cervical cancer cells EMT remodeling.

**Figure 5:**
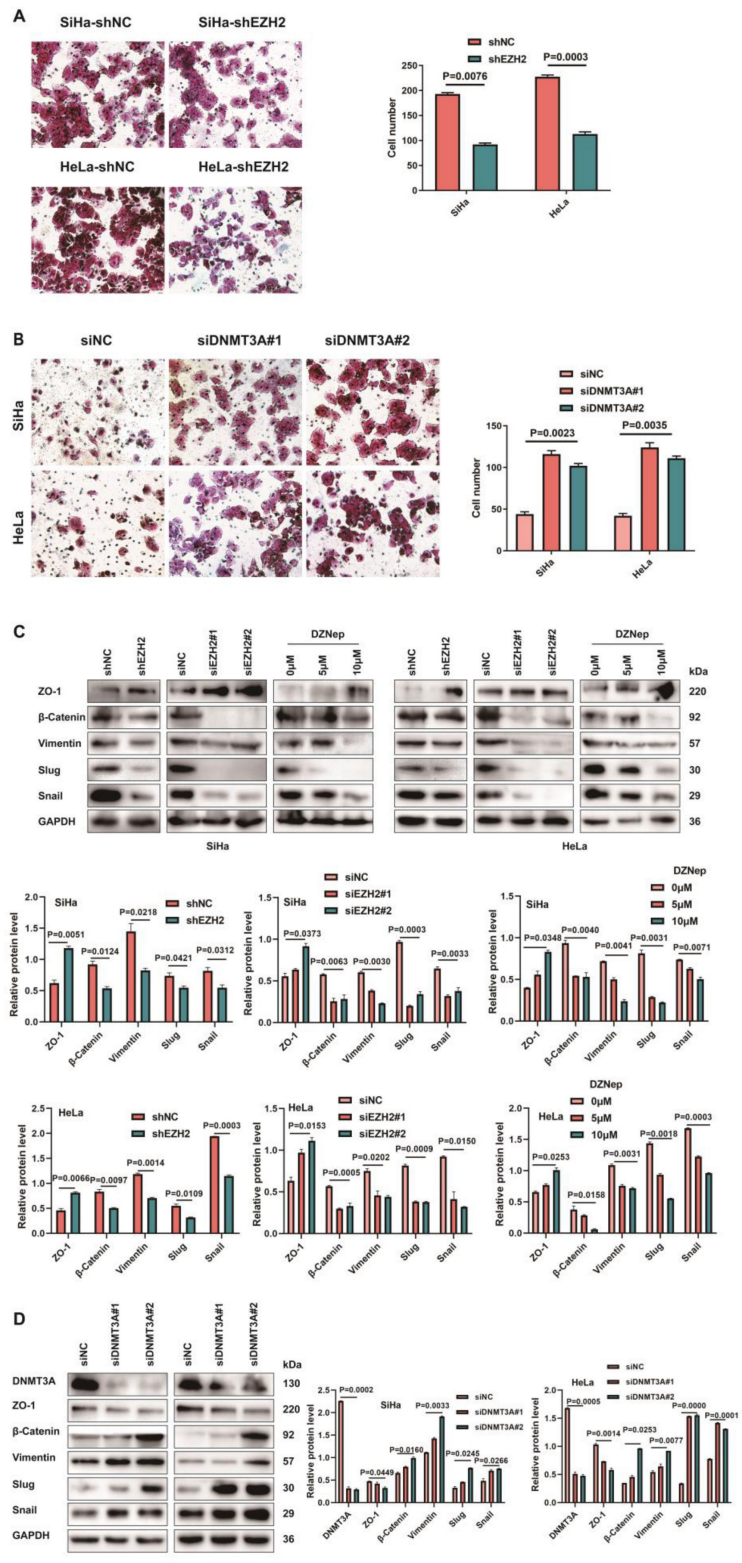
** Inhibition EZH2 represses migration in CC cells through downregulating CCL22-CCR4**. (**A**) The migratory potential of EZH2 knocked-down SiHa or HeLa cells and the respective control cells was analyzed by the transwell cell migration assay. Number of migratory cells was shown as means ± standard error from three independent experiments using triplicate measurements and statistically analyzed with Student's t-test in each experiment. Magnification, ×200. (**B**) The migratory potential of DNMT3A specific siRNA transfected SiHa and HeLa cells and the respective control cells was analyzed by the transwell cell migration assay. Number of migratory cells was shown as means ± standard error from three independent experiments using triplicate measurements and statistically analyzed with Student's t-test in each experiment. Magnification, ×200. (**C**) The expression of EMT-related proteins in EZH2 knocked-down or DZNep treated SiHa and HeLa cells were determined by western blotting and the gray level analysis of the protein levels of EMT-related proteins. (**D**) The expression of EMT-related proteins in DNMT3A specific siRNA transfected SiHa and HeLa cells were determined by western blotting and the gray level analysis of the protein levels of EMT-related proteins and DNMT3A.

CC cells with siRNA-mediated EZH2 knockdown displayed a lower migration capacity than the control cells. A weakened migration capacity was also seen when EZH2 was knocked-down by shEZH2 in SiHa and HeLa cell lines (Fig. 5A). To explore the mechanism of migration induced by EZH2, we investigated whether EZH2 could regulate EMT in CC cells by assessing vimentin, ZO-1, slug, snail and β-catenin, which are EMT hallmarks 20. Decreased vimentin, slug, snail and β-catenin levels and increased ZO-1 levels were observed in the siEZH2- and shEZH2-transfected groups. Moreover, CC cells treated with DZNep exhibited up-regulated expression of ZO-1 and downregulated vimentin, slug, snail and β-catenin protein levels (Fig. 5C). Knocking-down DNMT3A in CC cells led to increased migration capacity (Fig. 5B) and also changed expression of EMT-related markers (Fig. 5D) such as increased vimentin, slug, snail and β-catenin protein levels significantly and decreased ZO-1 expression.

**Figure 6:**
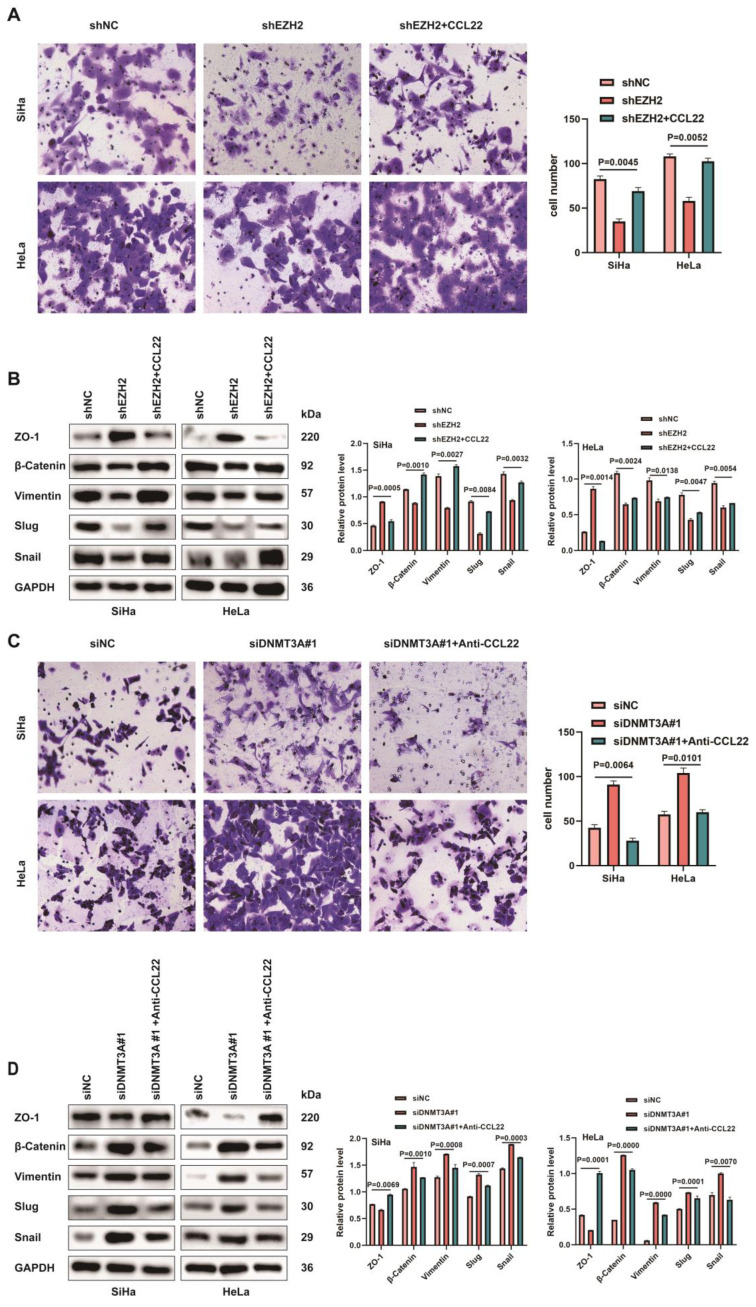
** CCL22-CCCR4 promotes migration in CC cells**. (**A**) The migratory potential of EZH2 knocked-down SiHa or HeLa cells with or without CCL22 and the respective control cells was analyzed by the transwell cell migration assay. Number of migratory cells was shown as means ± standard error from three independent experiments using triplicate measurements and statistically analyzed with Student's t-test in each experiment. Magnification, ×200. (**B**) The expression of EMT-related proteins in EZH2 knocked-down SiHa and HeLa cells with or without CCL22 were determined by western blotting. (**C**) The migratory potential of DNMT3A specific siRNA transfected SiHa and HeLa cells with or without anti-CCL22 and the respective control cells was analyzed by the transwell cell migration assay. Number of migratory cells was shown as means ± standard error from three independent experiments using triplicate measurements and statistically analyzed with Student's t-test in each experiment. Magnification, ×200. (**D**) The expression of EMT-related proteins in DNMT3A specific siRNA transfected SiHa and HeLa cells with or without anti-CCL22 determined by western blotting.

Next, we conducted transwell assay to further determine the involvement of CCL22-CCR4 in cell migration, which represented that the recombinant human CCL22 enhanced the ability of migration in CCR4-expressing shEZH2-transfected cells (Fig. 6A). Simultaneously, CC cells treated with recombinant human CCL22 exhibited downregulated expression of ZO-1 and upregulated vimentin, slug, snail and β-catenin protein levels (Fig. 6B). CCL22 neutralizing antibody treated with CCR4-expressing DNMT3A down-regulated CC cells inhibited the migration capacity (Fig. 6C), the expression of EMT-related markers reversed (Fig. 6D). The above results indicated that EZH2 induced migration and promoted EMT remodeling via CCL22-CCR4.

### EZH2-mediated epigenetic modulation of CCL22-CCR4 causing EMT remodeling *in vivo*

**Figure 7:**
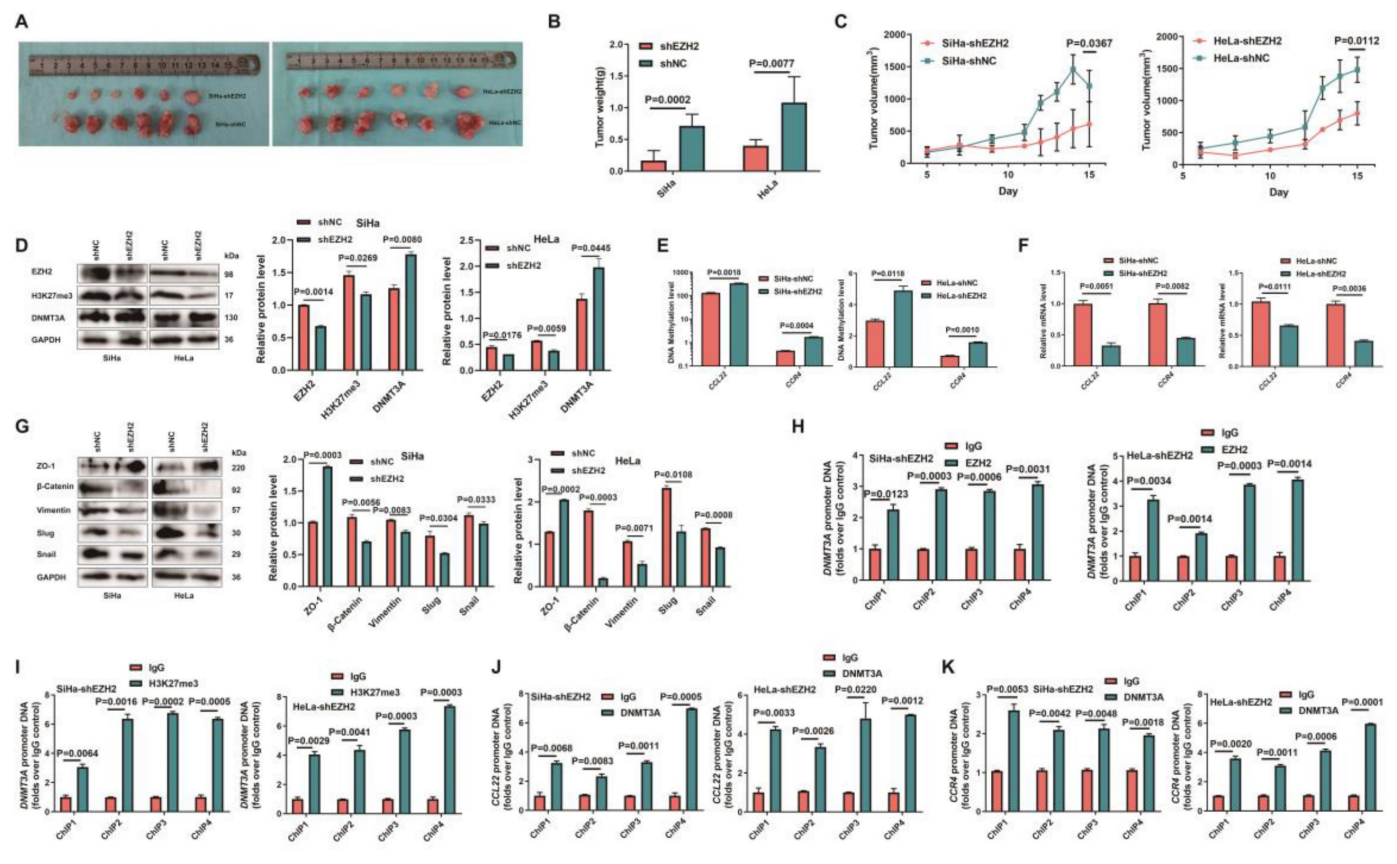
** Epigenetic modulation of CCL22-CCR4 mediated by EZH2* in vivo***. (**A**) SiHa-shEZH2 and HeLa-shEZH2 tumor xenografts in nude mice. (**B**) The tumors weight formed from SiHa-shEZH2 and HeLa-shEZH2. (**C**) Tumors formed from SiHa-shEZH2 and HeLa-shEZH2 cells as well as tumor growth curves. (**D**, **F** and **G**) Western blotting and RT-qPCR results of EZH2, H3K27me3, DNMT3A, CCL22-CCR4 and EMT-related proteins in SiHa-shEZH2 and HeLa-shEZH2 cells formed tumors. (**E**) The methylation level of *CCL22* and *CCR4* promoter regions were monitored by MS-qPCR in tumor tissues. (**H**-**K**) Chromatin was cross‑linked, fragmented and immunoprecipitated with either IgG (mock) or anti‑EZH2, H3K27me3 and DNMT3A ChIP‑grade antibody and the purified DNA was used to amplify with respective primer pairs for the indicated 4 regions in the *DNMT3A*, *CCL22*-*CCR4* promoters in qPCR. The enrichment of EZH2 and H3K27me3 on *DNMT3A*, *CCL22*-*CCR4* promoters and the enrichment of DNMT3A *CCL22*-*CCR4* promoters on relative to IgG in tumor tissues.

SiHa-shEZH2 and HeLa-shEZH2 tumor xenografts models in nude mice were established to investigate EZH2-mediated epigenetic modulation of CCL22-CCR4 *in vivo*. As shown in Fig. 7A, tumors derived from SiHa-shNC and HeLa-shNC cells (Fig. 7B and C) grew faster than those derived from SiHa-shEZH2 and HeLa-shEZH2 cells, respectively, indicating that downregulating EZH2 inhibited the growth of SiHa and HeLa cell-derived tumors.

To determine how EZH2 mediated CCL22-CCR4 expression through epigenetic regulation cause EMT remodeling* in vivo*, the levels of EZH2, H3K27me3, DNMT3A, EMT-related proteins and CCL22-CCR4 in xenograft tumor tissues were examined by western blotting or RT-qPCR. As shown in Fig. 7D, F and G, the expression of DNMT3A increased significantly when EZH2 was knocked down; CCL22 and CCR4 were downregulated in tumor tissues, and EMT-related protein expression also changed consistent with *in vitro* results. EZH2 knockdown significantly augment the methylation levels of* CCL22* and *CCR4* (Fig. 7E). ChIP analysis revealed that EZH2 regulated the expression of DNMT3A not only by directly binding to the* DNMT3A* promoter region but also by increasing H3K27me3 acting on the -1000 to +1 region of the promoter region of *DNMT3A* (Fig. 7H and I). DNMT3A was significantly enriched in the *CCL22* and *CCR4* promoter regions in tumor tissues (Fig. 7J and K). These results were consistent with those *in vitro*, illustrating that EZH2 mediated CCL22-CCR4 expression through epigenetic modulation so as to cause EMT remodeling *in vivo*.

## Discussion

Epigenetic alteration is essential for the carcinogenesis of cervical cancer, which results in the activation or exclusion of certain genes 21, 22. Studies on EZH2-mediated epigenetic changes and subsequent transcription changes have led to the development of precise cancer medicines 23-26. A previous study performed by the present authors revealed that EZH2 and H3K27me3 levels are higher and DNMT3A levels are lower in CC tissues compared with normal cervix 9. EZH2-dependent histone H3K27 trimethylation and DNA methylation via DNMTs have been found to lead to a cascade of events involving several coding and noncoding regions that ultimately result in glioblastoma aggressiveness 27. The present study identified DNMT3A as an important target of EZH2 and H3K27me3 in CC. EZH2 interacted with the *DNMT3A* promoter region and upregulated H3K27me3 levels in the *DNMT3A* promoter region in CC cell lines *in vitro*. Therefore, the increment of DNMT3A in CC cells after EZH2 knockdown or inhibition strongly indicated its silencing via EZH2-dependent H3K27me3 epigenetic mechanisms.

CCL22 is produced by cancer cells in tumors 28 and its expression is increased in colorectal adenocarcinomas 29. The expression of CCL22 is also elevated in breast cancer and associated with poor overall survival 30 and in the tumor microenvironment leading to a deterioration in the prognosis of patients with tongue squamous cell carcinoma 31. CCL22 could polarize tumor-associated macrophages of cervical cancer toward M2a macrophages 32. Patients with cervical cancer and elevated CCL22^+^ infiltrating cells require more aggressive treatment 33. CCL22 and its receptor CCR4 play an important role in homeostasis and inflammatory responses 34. Elevated CCL22 levels mediate the migration of CCR4-expressing Th2, which maintains the allergic process 35. CCR4 is a potential prognostic biomarker for the poor recurrence and survival of patients with pN0 oral tongue cancer, and CCR4 might be a possible therapeutic target for patients with early-stage cancer 36, 37. Previous findings of the present authors showed that *CCL22* and *CCR4* mRNA levels were higher in CC tissues compared with para-carcinoma tissue and normal cervix, and overexpression of *CCL22* and *CCR4* mRNA attributed immune disequilibrium in tumor microenvironment and promoted carcinogenesis 12. The regulatory mechanism of CCL22 and CCR4 in CC remains unclear. DNMT3A was found to interact with *CCL22* and* CCR4* promoter regions to enrich the methylation level of *CCL22* and *CCR4* promoter regions in CC cell lines. EZH2 knock-down or inhibition increased the DNA methylation and mRNA expression levels of the *CCL22* and *CCR4* promoter regions. EZH2-mediated the expression of DNMT3A, enhancing the DNA methylation levels of *CCL22* and *CCR4*. Hypomethylated promoter states of *CCL22* and *CCR4* eventually led to higher expression of CCL22 and CCR4 in CC tissues.

Tumor cells undergo the EMT which ultimately leads to metastasis and chemotherapy resistance 38. CCL22 was found to increases the proliferation of cancer cells 19, 39 and causes cancer cell migration and EMT in several types of cancers 19, 28, 40. The CCL22-CCR4 axis also participates in bone metastasis due to the high expression of CCL22 in bones 41. CCL22 significantly increased the migration of gastric cancer cells 42. Lung metastasis was also associated with CCR4 expression 43. Silencing CCL22 expression could reduce cell migration and invasion in head and neck cancer 44. The present study showed that the migration of CCR4-expressing CC cells was slowed when cells were treated with anti-CCL22 antibody. Moreover, vimentin, snail and slug expression levels were decreased, while ZO-1 expression was consistently increased. Increasing CCL22 levels increased the CCR4-expressing cancer cell migration rate, as well as vimentin, snail, slug, and β-catenin expression levels, in contrast, ZO-1 expression was decreased, suggesting that CCL22 combined with its receptor CCR4 could remodel the EMT process and promote the migration in CC cells. The present results also showed that a decrease in EZH2 suppressed the migration process of CC cells and led to the suppression of vimentin, snail, slug, and β-catenin expression and that increased ZO-1 levels are associated with increased EMT potential. Inhibiting CCL22-CCR4 to suppress the EMT of cervical cancer cells can attenuate distant metastasis and enhance the prognosis of patients with cervical cancer.

A novel EZH2 antagonist EIP103 was demonstrated to enter the nucleus, leading to pronounced cytotoxicity and significant anti-tumor activity in lung cancer 45. DNMT3A was indicated as a potential prognostic biomarker in glioma and a promising therapeutic target for treating patients with lower-grade glioma 46. In recent years, the incidence and mortality rates of cervical cancer in China have been increasing, with a notable trend towards younger age groups being affected, consequently, identifying precise targets for the treatment of cervical cancer is of paramount importance. The above findings indicate that EZH2 or DNMT3A and CCL22-CCR4 could serve as promising targets for the treatment of CC. Targeting EZH2 or DNMT3A and CCL22-CCR4 axis may impede the progression or metastasis of cervical cancer and enhance patient prognosis.

The present study is limited by the absence of further research on the values of EZH2 or DNMT3A and CCL22-CCR4 as potential therapeutic targets and prognostic biomarkers in CC. Additionally, the absence of *in vivo* validation experiments to illustrate that EZH2 facilitates tumor metastasis through epigenetic regulation of CCL22-CCR4 expression represents a limitation of this study. In future *in vivo* experiments, the combined inhibition of EZH2 and CCL22-CCR4 will be employed to investigate alterations in tumor proliferation, apoptosis and metastasis. Moreover, the limited availability of tumor tissue samples precluded further investigation into the association between CCL22 and CCR4 expression and tumor metastasis. The present authors will design experiments to verify these roles in the next research.

**Figure 8:**
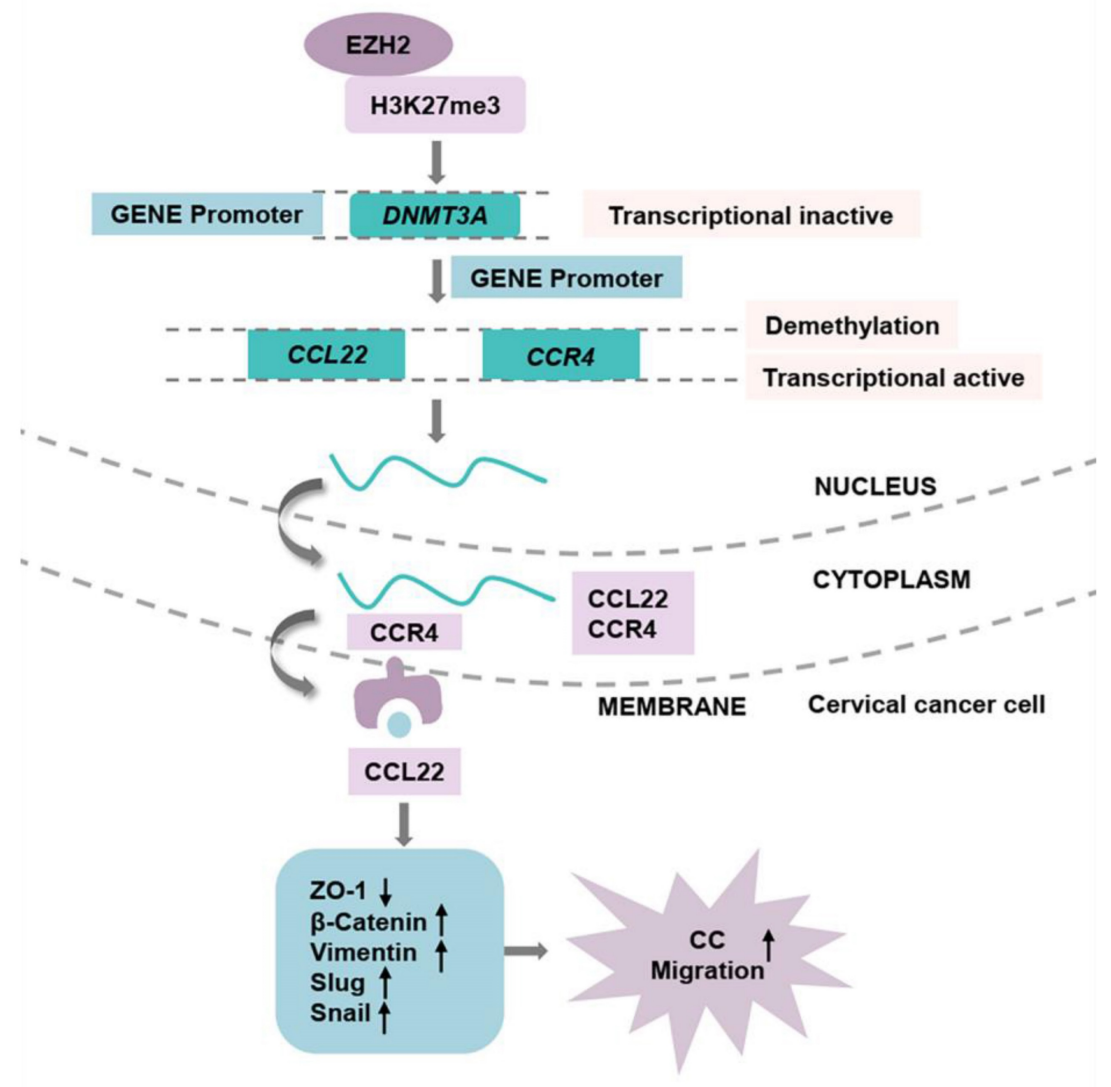
The pathway of EZH2 regulated CCL22-CCR4 expression through epigenetic modification causing EMT remodeling.

In conclusion, the current findings demonstrated that the higher expression of EZH2 regulates the higher production of CCL22 and CCR4 in CC. EZH2 promotes EMT process remodeling and migration through the binding of to CCR4 on CC cells. Knocking down EZH2 decreased H3K27me3 in the *DNMT3A* promoter region and altered the DNA methylation levels of *CCL22* and *CCR4* (Fig. 8). The epigenetic regulation driven by EZH2 plays a crucial role in the expression of CCL22-CCR4 and EMT in CC cells, thereby offering potential therapeutic targets for cervical cancer treatment.

## Figures and Tables

**Table 1:** Cancer patients' clinicopathological details (n = 32)

Item	No.
Age	
≤44	15
>44	17
FIGO stage	
ⅠA1-IB1	7
IB2-IIA2	16
IIB	4
ⅢC1	5
Pathological type	
Squamous cell carcinoma	28
Adenocarcinoma	4
Pathological grading	
Ⅰ	3
Ⅱ	22
Ⅲ	7
Tumor size	
<2 cm	11
≥2 cm	21
Lymph nodes metastasis	
Yes	5
No	27
HPV infection	
Positive	30
Negative	2

**Table 2:** Normal patients' clinicopathological details (n = 32)

Item	No.
Age	
≤44	10
>44	22
Disease	
Uterine myoma, fibroid	22
Adenomyosis	6
Ovarian cyst	3
Ovarian endometriotic cysts	1

**Table 3:** Primer sequences used in the study

Name	Application	Sequence
*CCL22*-ML	MS-PCR/MS-qPCR	GATAGGAGTGGGGGAGGGAGAGTTC
*CCL22*-MR	MS-PCR/MS-qPCR	AACCTATCTTATACCCTTAATAACG
*CCL22*-UL	MS-PCR/MS-qPCR	GATAGGAGTGGGGGAGGGAGAGTTT
*CCL22*-UR	MS-PCR/MS-qPCR	AACCTATCTTATACCCTTAATAACA
*CCR4*-ML	MS-PCR/MS-qPCR	TATAGTTGGGTGAGGGAGATAATTC
*CCR4*-MR	MS-PCR/MS-qPCR	ACCCAAAACCCTAAAAATATCCCCG
*CCR4*-UL	MS-PCR/MS-qPCR	TAGTTGGGTGAGGGAGATAATTTGT
*CCR4*-UR	MS-PCR/MS-qPCR	ACCCAAAACCCTAAAAATATCCCCA
*ALU*-F	MS-qPCR	TTAGGTATAGTGGTTTATATTTAGAATTTTAGTA
*ALU*-R	MS-qPCR	ATTAACTAAACTAATCTTAAACTCCATACCTCA
*EZH2*#1-sense	gene silencing	CGGCUUCCCAAUAACAGUATT
*EZH2*#1-anti-sense	gene silencing	UACUGUUAUUGGGAAGCCGTT
*EZH2*#2-sense	gene silencing	GACUCUGAAUGCAGUUGCUTT
*EZH2*#2-anti-sense	gene silencing	AGCAACUGCAUUCAGAGUCTT
*DNMT3A*#1-sense	gene silencing	GCCAAGGUCAUUGCAGGAATT
*DNMT3A*#1-anti-sense	gene silencing	UUCCUGCAAUGACCUUGGCTT
*DNMT3A*#2-sense	gene silencing	CCAUGUACCGCAAAGCCAUTT
*DNMT3A*#2-anti-sense	gene silencing	AUGGCUUUGCGGUACAUGGTT
Negative control-sense	gene silencing	UUCUCCGAACGUGUCACGUTT
Negative control-sense	gene silencing	ACGUGACACGUUCGGAGAATT
*CCL22*-F	RT-qPCR	GGTATTTGAACCTGTGGAATTGGAG
*CCL22*-R	RT-qPCR	CAGGCCCTGGATGACACTGA
*CCR4*-F	RT-qPCR	CCCTTAGGGATCATGCTGTT
*CCR4*-R	RT-qPCR	TCAAAGGTGCAGTCCTGAAG
*DNMT3A*-F	RT-qPCR	ATCTCCAAGTCCCCATCCA
*DNMT3A*-R	RT-qPCR	GTGCAGCAGCCATTTTCC
*EZH2*-F	RT-qPCR	TGGGAAAGTACACGGGGATA
*EZH2*-R	RT-qPCR	CAGGATCGTCTCCATCATCA
*GAPDH*-F	RT-qPCR	GCACCGTCAAGGCTGAGAAC
*GAPDH*-R	RT-qPCR	TGGTGAAGACGCCAGTGGA
*CCL22*-ChIP-F1	ChIP-qPCR	AAGTGACATTAAAGGCCAGGGACAG
*CCL22*-ChIP-R1	ChIP-qPCR	CTCCCCATTCACCTAAAGGCAGGTC
*CCL22*-ChIP-F2	ChIP-qPCR	GTTCTGAAGCAGGAGAGAGAGTGTG
*CCL22*-ChIP-R2	ChIP-qPCR	CTCAGATCTGCTCCTTCTCTCCAAC
*CCL22*-ChIP-F3	ChIP-qPCR	TAGGAAGCAGAATCAACGGGACATG
*CCL22*-ChIP-R3	ChIP-qPCR	TCGTGGATTTCTCAACGGTTTATGG
*CCL22*-ChIP-F4	ChIP-qPCR	AGACAGGCCTCAATTCTAGGTGAGG
*CCL22*-ChIP-R4	ChIP-qPCR	ATGTCTGGGTGTCTCTGGGACTTGG
*CCR4*-ChIP-F1	ChIP-qPCR	ATGACTGCTACCCACATCCAAAGTG
*CCR4*-ChIP-R1	ChIP-qPCR	GTGAAGTGGCCTGGCAACATAGCTT
*CCR4*-ChIP-F2	ChIP-qPCR	TTGAATCAAAGAATGTGGTTGGCTG
*CCR4*-ChIP-R2	ChIP-qPCR	GGGTGAGAAGGAAGGCCAGAGATAG
*CCR4*-ChIP-F3	ChIP-qPCR	CCCCTGCATATCCATGATGAGAAAC
*CCR4*-ChIP-R3	ChIP-qPCR	GGATTACAGGAATCAGCCACTCCTT
*CCR4*-ChIP-F4	ChIP-qPCR	AAAATTAGTCAGTCATGGTGGCTTG
*CCR4*-ChIP-R4	ChIP-qPCR	CTGTCACCCAGACTGGTGTTCAGTG

ChIP: chromatin immunoprecipitation, RT-qPCR: real-time quantitative reverse transcription polymerase chain reaction, F: forward primer, R: backward primer, MS-PCR: methylation-specific-polymerase chain reaction; ML/UL: methylation/unmethylation forward primer, MR/UR: methylation/unmethylation backward primer.

**Table 4:** Details of antibodies

Antibody	Source	Dilution	Cat Number	Application
EZH2	Cell Signaling Technology	1:100/1000/300	5246	ChIP, WB
H3K27me3	Cell Signaling Technology	1:50/1000/50	9733	ChIP, WB
DNMT3A	Abcam	1:50/250	ab13537	ChIP, WB
IgG	Cell Signaling Technology	1:500	2729	ChIP
Histone H3	Cell Signaling Technology	1:50	4620	ChIP
Vimentin	Cell Signaling Technology	1:1000	5741	WB
β-Catenin	Cell Signaling Technology	1:1000	8480	WB
ZO-1	Cell Signaling Technology	1:500	8193	WB
Snail	Cell Signaling Technology	1:500	3879	WB
Slug	Cell Signaling Technology	1:500	9585	WB
GAPDH	TransGen Biotech	1:500	HC301-01	WB
β-actin	TransGen Biotech	1:500	HC201-01	WB

ChIP=chromatin immunoprecipitation; WB=western blotting.
